# Willingness of veterinarians in Australia to recommend Q fever vaccination in veterinary personnel: Implications for workplace health and safety compliance

**DOI:** 10.1371/journal.pone.0198421

**Published:** 2018-06-01

**Authors:** Emily Sellens, Jacqueline M. Norris, Navneet K. Dhand, Jane Heller, Lynne Hayes, Heather F. Gidding, Harold Willaby, Nicholas Wood, Katrina L. Bosward

**Affiliations:** 1 Sydney School of Veterinary Sciences, Faculty of Science, the University of Sydney, Camperdown, NSW, Australia; 2 School of Animal and Veterinary Sciences, Charles Sturt University, Wagga Wagga, NSW, Australia; 3 National Centre for Immunisation Research and Surveillance, Westmead, NSW, Australia; 4 School of Public Health and Community Medicine, University of New South Wales Medicine, University of New South Wales, Kensington, NSW, Australia; 5 Sydney School of Public Health, the University of Sydney, Camperdown, NSW, Australia; 6 Discipline of Paediatrics and Child Health, Sydney Medical School, the University of Sydney, Camperdown, NSW, Australia; The University of Melbourne, AUSTRALIA

## Abstract

Q fever vaccine uptake among veterinary nurses in Australia is low, suggesting veterinarians are not recommending the vaccination to veterinary personnel. This study aimed to determine the willingness of veterinarians to recommend Q fever vaccination to veterinary personnel and to identify factors influencing Q fever vaccine uptake by veterinary nurses in Australia. An online cross sectional survey targeted veterinarians and veterinary nurses in Australia in 2014. Responses were analysed using multivariable logistic regression. Factors significantly (p<0.05) associated with a willingness to recommend the vaccination, expressed by 35% (95% CI 31–38%) of veterinarians (n = 828), were (1) being very concerned for colleagues regarding *Coxiella burnetii* (OR 4.73), (2) disagreeing the vaccine is harmful (OR 3.80), (3) high Q fever knowledge (OR 2.27), (4) working within small animal practice (OR 1.67), (5) disagreeing the vaccine is expensive (OR 1.55), and (6) age, with veterinarians under 39 years most likely to recommend vaccination. Of the veterinary nursing cohort who reported a known Q fever vaccination status (n = 688), 29% (95% CI 26–33%) had sought vaccination. This was significantly (p<0.05) associated with (1) agreeing the vaccine is important (OR 8.34), (2) moderate/high Q fever knowledge (OR 5.51), (3) working in Queensland (OR 4.00), (4) working within livestock/mixed animal practice (OR 3.24), (5) disagreeing the vaccine is expensive (OR 1.86), (6) strong reliance on work culture for biosecurity information (OR 2.5), (7) perceiving personal exposure to *Coxiella burnetii* to be at least low/moderate (OR 2.14), and (8) both agreeing the vaccine is safe and working within a corporate practice structure (OR 4.28). The study identified the need for veterinarians to take greater responsibility for workplace health and safety promotion, and calls for better education of veterinary personnel to raise awareness of the potential for occupational exposure to *C*. *burnetii* and improve the perception of the Q fever vaccine as being important, safe and cost-effective.

## Introduction

Q fever is a vaccine-preventable zoonotic disease caused by the bacterium *Coxiella burnetii*, which is distributed worldwide, with the exception of New Zealand and French Polynesia [1]. Although acute illness is usually limited to non-specific flu-like symptoms, up to five percent of patients experience severe illness requiring hospitalisation and one percent of acute clinical cases are fatal [2–7]. Patients with cardiovascular lesions, immunosuppression or pregnancy are predisposed to persistent focalized *C*. *burnetii* infections, such as endocarditis, vascular infections, and bone and joint infections [1]. Infection during pregnancy may lead to adverse pregnancy outcomes including miscarriage [1, 7]. Diagnosis is often delayed in the absence of suspicion; highlighting the importance of prevention in at-risk cohorts [8, 9].

Farmed cattle, sheep and goats are most commonly implicated in human Q fever [5], however human outbreaks associated with dogs and cats are well described [3, 10–12] and *C*. *burnetii* has been found within a wide range of host species [5, 13]. Bacteria are shed in greatest numbers from the placenta of infected animals at parturition, while chronic shedding may occur in the urine, faeces and milk [5]. Coxiellosis may manifest as abortion, still birth and low birth weight in cattle, sheep and goats, while clinical manifestations in other species is poorly understood and not well documented [14]. However, the majority of infected animals remain asymptomatic during both acute and persistent infection, and coxiellosis is rarely diagnosed in animals in the absence of routine surveillance [5].

*Coxiella burnetii* is highly resistant and capable of remaining viable in the environment for at least 12 months [15]. Inhalation is the major route of transmission to humans and the bacterium may be spread over long distance by wind and in dust [5]. People exposed to animals are considered at greatest risk of exposure to *C*. *burnetii*, and Q fever is subsequently a workplace health and safety concern for farmers, meat processors, and veterinarians globally [16]. Military personnel deployed to the Middle East are also at high risk of infection due to the prevalence of *C*. *burnetii* in region [17]. While personal protective equipment offers some protection during risky procedures, human vaccination is the most effective measure for preventing Q fever and related societal costs.

Q fever is associated with considerable costs to individuals, businesses, and public health systems; including medical expenses, lost work hours and compensation claims. A large outbreak which occurred in the Netherlands from 2007–2011 is estimated to have cost over 300 million Euros, with delayed expenses associated with chronic fatigue syndrome an ongoing prominent burden [18]. In Scotland, an outbreak among abattoir workers in 2006 highlighted the burden and complexities of managing outbreaks and the long term follow up of affected workers [19]. Within Australia, Q fever is the most commonly reported notifiable zoonotic disease, excluding food-borne pathogens [20]. Here, the cost of compensation alone is estimated to exceed $1.3 million Australian dollars annually [21]. The true cost of this disease is likely to be underestimated, as many cases remain un-diagnosed where there is a lack of suspicion [21].

A whole-cell Q fever vaccine (Q-Vax; Seqiris, Parkville, Vic.) has been available in Australia since 1989 and is recommended for workers with high occupational risk; including veterinary workers [22]. Targeted Q fever vaccination of people within high-risk occupations was shown to significantly reduce the burden of Q fever in Australia and proved to be cost-effective [21, 23]. The European Centre for Disease Prevention and Control has called for the use of the Australian vaccine in Europe until a new generation vaccine is developed [24], and the vaccine was subsequently implemented in the Netherlands in response to the large Q fever outbreak [25, 26]. Despite this, Australia remains the only country to routinely use a human Q fever vaccine. Development of an effective new generation vaccine has been unsuccessful to date, however research is ongoing.

Uptake of the Q fever vaccine among at-risk workers in Australia is variable. Uptake by abattoir workers and sheep shearers targeted in a nation-wide government funded vaccine program ranged from 50–100% across different Australian states [23]. Uptake by veterinarians, who since the early 1990s have been mostly vaccinated *en mass* upon commencing university studies, is estimated at 74% [27]. In contrast, a best-case estimate of 29% for uptake among veterinary nurses was reported [27], with the low uptake attributed to a lack of awareness of the Q fever vaccine, a lack of knowledge regarding Q fever disease, and an increase in the influence of barriers to vaccination where vocational vaccine programs or protocols are not routine [27]. The poor vaccine uptake among veterinary nurses may also be due to veterinarians, who are a major influential source of biosecurity information for this cohort [27], not recommending, or insisting on, Q fever vaccination in veterinary personnel under their employment. This raises serious concerns regarding workplace health and safety (WH&S) within the Australian veterinary industry.

This study aimed to determine the willingness of veterinarians in Australia to recommend Q fever vaccination to other veterinary personnel and to identify significant factors influencing the uptake of the Q fever vaccine by veterinary nurses in Australia. The results of this study have the potential to inform WH&S and Q fever vaccination protocols within the Australian veterinary industry and provide valuable knowledge for the implementation of Q fever vaccination internationally.

## Methods

### Study design and recruitment

An online cross sectional survey targeting all actively working veterinarians and veterinary nurses in Australia was undertaken from March to June of 2014via the Survey Monkey platform. The details of this study and recruitment have been described previously [27]. Briefly, the questionnaire contained 53 questions pertaining to (1) demographics and veterinary work environment, (2) attitudes towards Q fever illness and vaccination, (3) experience with Q fever disease, (4) experience with Q fever vaccination, (5) knowledge of disease risk, and (6) biosecurity practices.

Veterinary nurses in Australia are not required to be formally registered with state veterinary boards, with the exception of those in Western Australia. Veterinarians however, are required to maintain registration with the veterinary board of the state in which they practice. A personal email invitation to participate in this survey was sent on our behalf to veterinary nurses in Western Australia, and veterinarians in Western Australia and Tasmania, from their respective state veterinary boards. Elsewhere, contact via state veterinary boards was not possible and participants were primarily recruited via their workplace. Researchers attempted to phone all veterinary clinics in Australia to invite participation and a follow up invitation was sent via email, or fax or post where preferred, to consenting clinics to be shared with staff. Additionally, the survey was advertised by (1) the Australian Veterinary Association in an e-newsletter distributed to members on the 11^th^ of April 2014, (2) the Veterinary Practitioners Board of New South Wales on their website during May 2014, and (3) the Veterinary Nursing Council of Australia (VNCA) as a personal email invitation to members.

The study was performed in accordance with the Declaration of Helsinki. A participant information statement outlining the risks and benefits of the study was provided to participants upon invitation, and again in the first pages of the survey. Consent to participate was confirmed through commencement of the survey. Ethics approval was granted by Charles Sturt University School of Animal and Veterinary Sciences Human Ethics Committee (protocol #416/2013/19).

### Data management and analysis

#### Outcome variables

**Fig 1:**
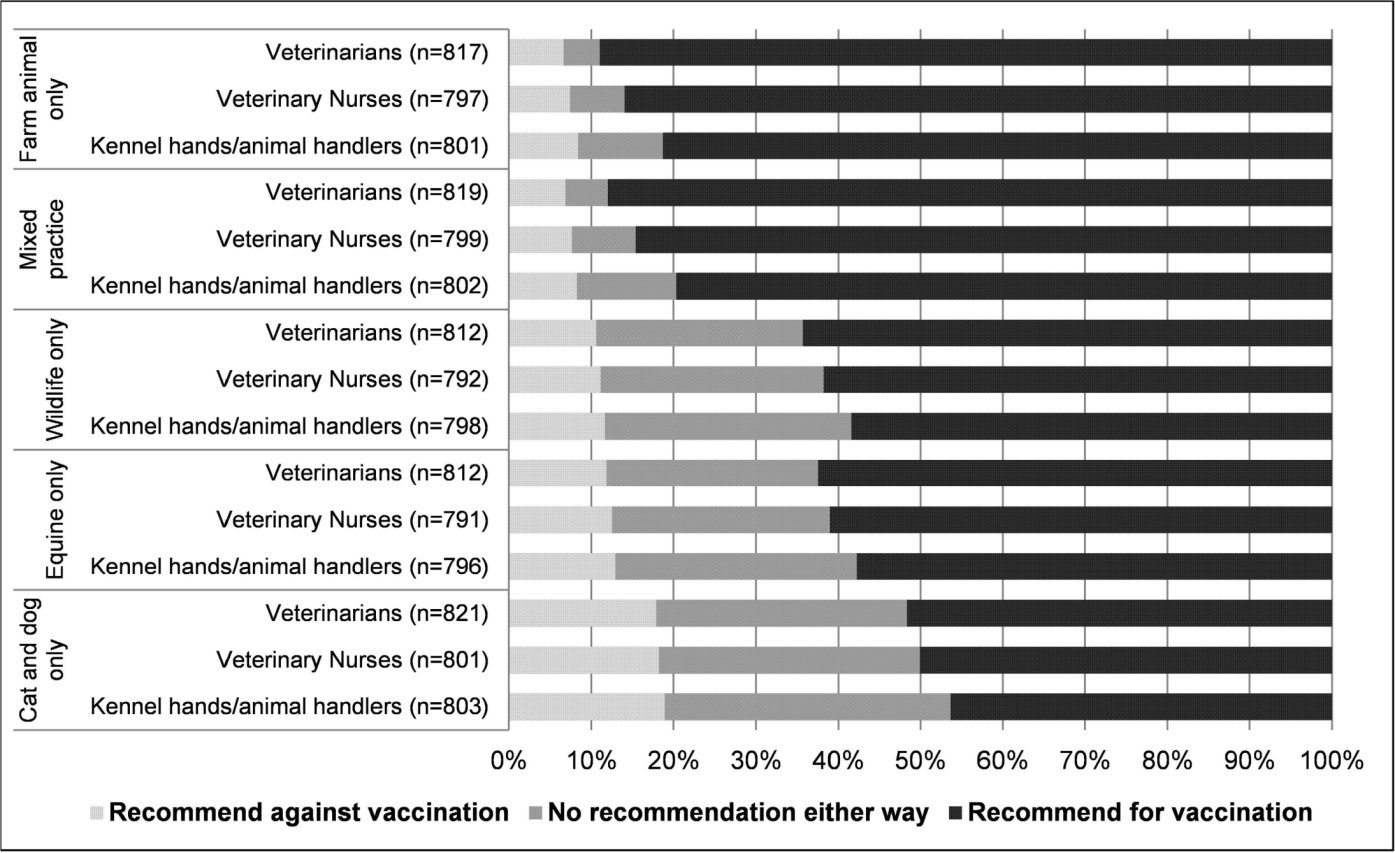
Responses from veterinarians (n = 828) surveyed in Australia in 2014 reflecting their recommendation for Q fever vaccination in veterinary personnel. The percentage of veterinarians recommending for vaccination, against vaccination, and with no recommendation either way for veterinary occupational groups (veterinarians, veterinary nurses, kennel hands/ animal handlers) is presented for each practice type queried. Not all respondents provided a recommendation for all occupations across all practice types.

Two outcome variables were drawn from the questionnaire data. A dichotomous outcome variable was created for veterinarians from responses to the survey question “*Thinking about vaccination for Q fever across each occupation group within each practice type*, *what would be your recommendations for Q fever vaccination*?*”* (Fig 1). Veterinarians who indicated that they slightly, moderately or strongly recommended vaccination for veterinarians, veterinary nurses and kennel hands/animal handlers across all veterinary practice types were considered ‘willing to recommend vaccination’.

A second dichotomous outcome variable reflecting vaccination status was assessed for the veterinary nursing cohort. Those nurses who were vaccinated, or had attempted vaccination but were unable to receive the vaccine due to a positive pre-vaccination screening result, were considered to have ‘sought vaccination’ which was the positive outcome variable. Veterinary nurses who stated ‘unsure’ for their vaccine status were excluded from the statistical analysis.

#### Explanatory variables

For the outcome variables ‘willing to recommend Q fever vaccination’ and ‘sought vaccination’, 38 and 33 explanatory variables were assessed respectively (S1 and S2 Tables). Age, gender, state, practice type, practice structure, and education were considered potential confounders.

The continuous variables ‘age’, ‘years working’ and ‘self-rated Q fever knowledge’ were categorized into ordinal variables for each cohort determined by their distribution into quantiles. Practice type was defined by the proportion of hours respondents spent per week with each species: (a) ‘small animal’ where >90% of work hours were spent with cats, dogs, pocket pets, wildlife or birds; (b)‘equine’ where >90% of work hours were spent with horses; (c)‘livestock’ where >90% of work hours were spent with cattle, sheep or goats; (d) ‘other’ where >90% of work hours were spent with zoo, fish, or other species and the remainder were classified as (e) ‘mixed’ animal practice. Due to the low number of veterinary nurses working in some practice types, a dichotomous variable was created; ‘livestock/mixed animal practice’ reflecting exposure to farm animals which are most commonly implicated as sources of Q fever in Australia, and ‘small/equine/wildlife/other’ reflecting exposure to species that are less commonly implicated in Q fever cases in Australia [28, 29]. Participant’s practice structure was determined by the practice environment in which most weekly hours were spent. Hours were stated for (a) solo practice (single vet), (b) group/multi-vet practice (c) corporate practice (group practices owned by a large corporation), (d) government (e) industry (f) laboratory (g) university (h) abattoir and (i) other. Due to the small number of participants working outside of solo and group practices and the similarities in general management styles of practice structures (c) through to (i), the latter were combined as one practice structure labelled “corporate/other”.

#### Statistical analysis

Initially, contingency tables and univariable associations between explanatory and outcome variables were determined, assisted by UniLogistic SAS macro [30]. Variables exhibiting some association (p<0.25) were then considered for multivariable logistic analysis, excluding those for which >10% of responses were missing. Collinearity was assessed using the Spearman rank correlation coefficient and Chi-square test of significance. Where two variables were found to be collinear (coefficient >0.7; p-value <0.05) one of the pair of collinear variables was excluded from multivariable analysis. Multivariable model building, aided by MultiLogistic SAS macro [31], was undertaken via forward stepwise selection retaining variables with a p-value <0.05 in the final model.

All significant variables within the model and potential confounders were tested for interaction prior to assessment of confounding. Interaction terms were retained in the model where significant (p<0.05), and potential confounders were forced into the model if they caused >20% change in the coefficients of variables already in the model. The Likelihood-ratio test was used to determine the significance of the full models and Hosmer-Lemeshow goodness of fit tests were performed on final models.

## Results

### Sampling

Eligible responses from 1,742 participants were received; 890 veterinarians and 852 veterinary nurses. This resulted from telephone contact with 1,677 clinics, of which 1,446 and 1,582 consented to receive the survey for participation of veterinary nurses and veterinarians respectively. Additionally, personal invitation emails sent to 882 veterinary nurses and 1200 veterinarians registered with the Western Australia state veterinary board, 245 veterinarians registered with the veterinary board of Tasmania, and 917 veterinary nurse member of the VNCA.

It was not possible to calculate a response rate for the survey as the number of veterinarians and veterinary nurses that viewed an invitation to participate is not known. Referring to government employment statistics however, the number of responses represented 12% of the estimated 7,400 employed veterinarians and 10% of the estimated 8,600 employed veterinary nurses in Australia at the time [32, 33]. Further results of sampling, including the characteristics and demographics of the study sample have been previously described in detail [27].

### Willingness to recommend Q fever vaccination

Of the 890 veterinarians who participated, 828 responses were complete for the variable ‘willing to recommend vaccination’. Of these, 287 (35%; 95% confidence interval [CI] 31–38%) were considered willing to recommend Q fever vaccination. Generally, a greater proportion of veterinarians were willing to recommend Q fever vaccination to workers in livestock and mixed animal practices than other practice types (Fig 1).

Of the 38 explanatory variables assessed, 11 were excluded due to >10% missing data. The first reflected self-rated biosecurity knowledge and the low response rate is attributed to respondents visually missing the question during survey completion. The further ten excluded variables pertained to sources of biosecurity information, and survey fatigue is likely responsible for the poor responses as all were drawn from the final question of the survey. Of the remaining 27 variables, 19 exhibited some univariable association (p<0.25) with the outcome (S1 Table). Four were excluded due to significant correlation with other variables and the remaining 15 variables were tested in the multivariable analysis.

Multivariable modelling identified six variables significantly associated with the outcome ‘willing to recommend vaccination’; (1) level of concern that colleagues may be exposed to *C*. *burnetii*, (2) concern that the Q fever vaccine will do more harm than good, (3) age, (4) self-rated Q fever knowledge, (5) gender, and (6) perception of vaccine expense. No significant interaction terms were identified. Practice type was found to confound the association between the outcome and three of the significant variables; (1) level of concern that colleagues may be exposed to *C*. *burnetii* and (2) self-rated Q fever knowledge and (3) gender. The inclusion of practice type rendered gender non-significant; however gender was retained in the model as it confounded the association of age with the outcome. The Hosmer and Lemeshow Goodness-of-Fit test was not significant (p = 0.59) demonstrating there was no reason to believe the model was not a good representation of the data.

The final multivariable model included 772 complete case responses of which 270 (35%) were willing to recommend the Q fever vaccination. Veterinarians willing to recommend Q fever vaccination were more likely to; (1) report higher levels of concern that colleagues may be exposed to *C*. *burnetii*, (2) disagree that the Q fever vaccine will do more harm than good, (3) work in small animal practice, (4) self-report higher levels of Q fever knowledge, (5) be under 39 years of age, and (6) disagree that Q fever vaccination is expensive (Table 1).

**Table 1:** Final multivariable model parameter estimates and odds ratios of factors associated with a willingness to recommend Q fever vaccination among veterinarians surveyed in Australia in 2014.

Variable	b	SE(b)	Odds Ratio	95% LCL	95% UCL	P-value^a^
**Intercept**	-3.61	0.5	.	.	.	<0.001
**Concern that colleagues may be exposed to *C*. *burnetii***						<0.001
Nil concern	.	.	Ref	.	.	
Slight concern	0.89	0.23	2.43	1.54	3.87	
Moderate concern	1.5	0.26	4.47	2.71	7.46	
Very concerned	1.53	0.32	4.62	2.47	8.74	
**"I worry that the Q fever vaccine will do more harm than good"**						<0.001
Agree	.	.	Ref	.	.	
Disagree	1.4	0.38	4.05	2.02	9.07	
**Practice type in which most hours have been spent throughout career**						0.008
Mixed/Large animal	.	.	Ref	.	.	
Small animal	0.51	0.19	1.67	1.16	2.42	
Other	0.68	0.44	1.97	0.82	4.59	
**Self-rated Q fever knowledge**						0.019
≤3/10	.	.	Ref	.	.	
4/5/2010	0.2	0.24	1.22	0.76	1.95	
6/7/2010	0.6	0.24	1.82	1.14	2.92	
≥8/10	0.79	0.29	2.2	1.24	3.92	
**Age**						0.019
≤30 years	.	.	Ref	.	.	
31–38 years	-0.12	0.22	0.89	0.58	1.38	
39–48 years	-0.69	0.24	0.5	0.31	0.8	
≥49 years	-0.48	0.25	0.62	0.37	1.01	
**"The Q fever vaccination is too expensive"**						0.039
Agree	.	.	Ref	.	.	
Disagree	0.44	0.19	1.55	1.07	2.27	
Don't know	0.03	0.24	1.03	0.64	1.65	

Model adjusted for Gender. b; regression coefficient. SE; standard error. OR; profile-likelihood odds ratio. LCL; 95% lower confident limit. UCL; 95% upper confidence limit. Ref; Reference category.

^a^Likelihood ratio P-value.

### Factors influencing vaccine uptake by veterinary nurses

Of the 852 veterinary nurse respondents, 729 entered a response for vaccination status. Those stating ‘unsure’ (n = 41) were excluded from further analysis. Of the 688 remaining veterinary nurses, 199 (29%; 95% CI 26–33%) had sought vaccination for Q fever [27]. Of 33 explanatory variables assessed, one was excluded due to >10% missing data; self-rated knowledge of biosecurity. This was attributed to respondents visually missing the question during survey completion. Twenty-five of the remaining 32 variables exhibited some univariable association (p<0.25) with the outcome (S2 Table). Four were excluded due to significant correlation with other variables and the remaining 22 variables were included in multivariable analysis.

The final multivariable model included 573 complete case responses of which 166 (29%) had sought vaccination. Nine main effects were identified (Table 2). Interaction was found between perception of Q fever vaccine safety and practice structure in which most hours were spent (Table 2). Age and gender were forced into the model as significant confounders. Age confounded the association between perception of Q fever vaccine safety and the outcome, while gender confounded the association of practice structure with the outcome. The Hosmer and Lemeshow Goodness-of-Fit test was not significant (p = 0.23) demonstrating there was no reason to believe the model was not a good representation of the data.

**Table 2:** Final multivariable model parameter estimates and odds ratios of factors significantly associated with Q fever vaccination status of veterinary nurses in Australia in 2014.

Variable		b	SE(b)	Odds Ratio	95% LCL	95% UCL	P-value^a^
**Intercept**		-4.09	0.98	.	.	.	0.009
**"I am convinced of the importance of the Q fever vaccination"**							0.001
Disagree		.	.	Ref	.	.	
Agree		2.12	0.8	8.34	2.16	56.35	
**Self-rated Q fever knowledge**							<0.001
1/10		.	.	Ref	.	.	
2-3/10		0.58	0.44	1.78	0.78	4.36	
4-5/10		1.17	0.45	3.21	1.37	8.05	
6+/10		1.71	0.47	5.51	2.28	14.29	
**State**							<0.001
WA / NT		.	.	Ref	.	.	
SA / Tasmania / Victoria		0.43	0.5	1.54	0.59	4.24	
NSW / ACT		0.22	0.47	1.24	0.51	3.25	
Queensland		1.39	0.49	4	1.59	10.81	
**Practice type**							0.001
Small / equine / wildlife / other		.	.	Ref	.	.	
Large / mixed		1.18	0.3	3.24	1.8	5.93	
**"The Q fever vaccination is too expensive"**							<0.001
Agree		.	.	Ref	.	.	
Disagree		0.62	0.31	1.86	1.01	3.45	
Don't know		-0.55	0.29	0.58	0.33	1.02	
**Reliance on work culture for biosecurity information**							0.022
Nil		.	.	Ref	.	.	
Minor / moderate		0.33	0.4	1.39	0.65	3.12	
Major / sole		0.92	0.41	2.5	1.15	5.69	
**Perceived average personal level of exposure to *Coxiella burnetii* throughout career**							0.028
Nil/very low		.	.	Ref	.	.	
Low/moderate		0.76	0.27	2.14	1.26	3.66	
High/very high		1.07	0.65	2.91	0.83	10.85	
Don't know		0.16	0.37	1.17	0.56	2.41	
***Interaction Term***							
**"The Q fever vaccination is safe if appropriately administered"**	**Practice structure in which most hours are spent**						0.032
Disagree / don't know	Solo	.	.	Ref			
	Group	.	.	0.15	0.02	0.87	
	Corporate / other	.	.	0.36	0.04	2.33	
Agree	Solo	.	.	Ref			
	Group	.	.	1.14	0.66	1.99	
	Corporate / other	.	.	4.28	1.87	10.15	

Positive outcome = 'sought vaccination'. Model adjusted for age and gender. b; regression coefficient. SE; standard error. OR; profile-likelihood odds ratio. LR; likelihood ratio test. LCL; 95% lower confidence limit. UCL; 95% upper confidence limit. Ref; reference category. NSW; New South Wales. ACT; Australian Capital Territory. SA; South Australia. WA; Western Australia. NT; Northern Territory.

^a^Likelihood ratio P-value.

Veterinary nurses who had sought vaccination were more likely to; (1) be convinced of the importance of the Q fever vaccination, (2) self-report higher levels of Q fever knowledge, (3) work in Queensland, (4) work with animal species more commonly associated with Q fever, (5) disagree that the vaccine is expensive, (6) rely mostly or solely on workplace culture as a source of biosecurity information, (7) report greater likelihood of exposure to *Coxiella burnetii*, and (8) both agree that Q fever vaccination is safe and work in corporate/ other practice structures.

## Discussion

According to Australian law, workers must show due diligence to ensure the health and safety of themselves and others within their workplace, including eliminating or minimising WH&S risks as far as is reasonably practicable and providing necessary training and instruction to protect all persons [34]. Failure to comply can result in legal action against business owners or employees, and hefty workers’ compensation claims [34]. Regarding Q fever, it could be argued that the first WH&S priority should be the recommendation of Q fever vaccination by veterinarians, who have specific knowledge on public health and zoonoses, to other veterinary personnel. This study however, identified that only 35% of veterinarians surveyed in 2014 demonstrated some level of willingness to recommend the Q fever vaccination to veterinary personnel across all practice types.

Small animal veterinarians and veterinarians reporting high Q fever knowledge scores were most likely to recommend Q fever vaccination in veterinary personnel across all practice types. The finding that veterinarians associated with livestock and mixed animal practice were less likely to recommend vaccination across all practice types reflects a misunderstanding of the relevance of Q fever across all veterinary practice types and to all employed veterinary personnel. As Q fever is most often associated with ruminants in Australia and many places worldwide, veterinarians working with these species and veterinarians with low Q fever knowledge may not identify that other species pose a threat of Q fever. This is further supported by the decreased vaccine uptake reported by veterinary nurses working with species not traditionally associated with Q fever.

With cases of Q fever being reported among small animal workers both within Australia and abroad [10–12], it is essential that veterinary personnel acknowledge that all animal species, and particularly periparturient animals, pose a potential threat of Q fever. Improving the understanding of the risk of exposure to *C*. *burnetii* for all veterinary personnel should improve both vaccination recommendation and uptake. This is further supported by the findings that increased concern for colleagues regarding exposure to *C*. *burnetii* was associated with recommendation by veterinarians, and veterinary nurses reporting higher Q fever knowledge and those perceiving at least a low/moderate level of exposure to *C*. *burnetii* were more likely to take up Q fever vaccination.

Age was found to be a significant factor for recommendation of Q fever vaccination, with veterinarians aged less than 39 years more likely to recommend the vaccination. This may reflect improvements over time in the teaching of public health at a tertiary level, the introduction of strict Q fever vaccination protocols within Australian veterinary schools in recent years, and vaccine availability from 1990 onwards.

Concern that the Q fever vaccine may be harmful significantly decreased the odds of veterinarians recommending the vaccine. Although the vaccine results in local injection site reactions in up to 80% of vaccinees [35–37], serious adverse events are extremely rare due to strict pre-vaccination protocols including serological and intradermal skin testing to screen for pre-existing immunity [36–38]. Both the skin test and vaccine have been proven very safe [36] and efficacy is reported to be greater than 97% [38, 39]. The notion that this vaccine is likely to be harmful when administered appropriately is unfounded, and overcoming this perception through education and awareness should help to improve vaccine recommendation.

Although the pre-vaccination screening process markedly improves safety, it does result in a more complicated, time consuming and expensive vaccination process. This study identified that the perception that the Q fever vaccine was expensive was a significant factor associated with reduced vaccine recommendation by veterinarians and vaccine uptake among veterinary nurses. Vaccine expense could potentially be decreased through group vaccination and vaccine subsidies from the government or employers; strategies which have proven to increase vaccine uptake and reduce disease burden among other at-risk cohorts [23]. Improved perception of cost-benefits could also be achieved through education of veterinary nurses to improve Q fever knowledge and convince them of the importance of Q fever vaccination. Veterinary and veterinary nursing conferences should include continuing education on Q fever and provide tools to help veterinary managers implement Q fever vaccination protocols and programs within clinics. Veterinary nurses who reported workplace culture as a major influence regarding biosecurity practices were more likely to have sought Q fever vaccination. This finding implies that veterinary workplaces in which organisational culture places a high level of importance on safety foster positive attitudes towards Q fever vaccination. This is further supported by the positive association with having sought vaccination and employment within corporate practices and other organizations including abattoirs, government facilities and universities. These employers tend to have formal business-like structures with clear WH&S policies and procedures underpinning workplace safety culture; attributes that have been shown to positively impact the uptake of occupational vaccines among other healthcare workers [40–42]. Unfortunately WH&S attitudes and practices are typically inadequate in the majority of Australian veterinary practices [43] and the findings of this study provide further evidence that a change in WH&S culture is required within the industry as a whole.

Veterinary nurses working in the state of Queensland reported significantly higher odds of vaccination than other Australian states. This is not surprising given that it was Queensland in which the first case of Q fever was described in 1935 [44], and that the state has the highest annual Q fever notification rate in Australia [20]. However, Q fever is present in all states of Australia and efforts to improve vaccine uptake are required on a national level [45]. A unified approach across national veterinary bodies, such as the Australian Veterinary Association and the Veterinary Nursing Council of Australia, and state veterinary boards could potentially be beneficial in promoting Q fever vaccination. Consideration of compulsory national registration for veterinary nurses would improve access to this occupational group for the purposes of providing accurate WH&S information.

Limitations of this study relating to accessing this unique workforce, response rates, sample representativeness, and selection bias have been discussed in detail previously [27]. Briefly, access to the veterinary workforce proved difficult, particularly the veterinary nursing cohort who are not subject to strict registration requirements. A novel approach was required to ensure contact with as many veterinary workers as possible; however this led to an unknown denominator for calculation of response rates. Alternative methods, such as data collection at professional conferences, would have allowed for accurate response rates but resulted in much smaller sample sizes and greater selection bias. Given the limitations, the sample size achieved in this study was exceptional and the cohort of veterinarians was considered representative of the population when compared to government employment and Australian Veterinary Association statistics [32, 46].

Selection bias towards participation from those familiar with Q fever vaccination may have contributed to these results representing a ‘best-case’ scenario for vaccine recommendation and uptake. For the veterinary nursing cohort, the exclusion of veterinary nurses stating ‘unsure’ for their Q fever vaccination status would have compounded this bias, as the authors propose that these participants were most likely not vaccinated as this vaccine is relatively ‘memorable’ given the complexity of vaccination and frequency of local adverse reactions. However, exclusion of this small proportion of respondents is not likely to have affected the outcome of regression analysis. Importantly, to be considered willing to recommend vaccination, veterinarians in this study had to at least slightly recommend the vaccination to veterinarians, veterinary nurses and kennel hands across all practice types queried. Ideally, veterinarians should be strongly recommending the Q fever vaccination to all veterinary personnel. Additionally, the veterinary nursing cohort responding to the questionnaire was likely to be older and more highly educated than the veterinary nursing population as a whole [27, 33], indicating that a bias towards participation by those more familiar with the topic of Q fever may exist. As such, the proportion of veterinarians willing to recommend vaccination and veterinary nurses taking up vaccination represent a best-case scenario for this workforce, with the need for improvement within the industry probably being even more pressing than highlighted by these study results.

Internationally, the results of this study should be considered for any proposed Q fever vaccination programs utilising Q-VAX or new generation vaccines should they become available. To date, routine use of Q-VAX has been limited to occupational cohorts within Australia. The vaccine was successfully implemented for a short time in the Netherlands, however vaccination was targeted towards patients pre-disposed to Q fever complications; mostly elderly people with immunosuppressive, cardiac, or vascular disease [25, 26, 36]. As the outbreak subsided, the vaccination program was discontinued and ongoing prevention of human Q fever instead focussed on vaccination of livestock to limit shedding of *C*. *burnetii* [18]. Barriers to the routine use of Q-VAX in the Netherlands and elsewhere focus largely on the complexities and expense of the pre- vaccination screening process and the reactogenicity of the vaccine [17, 23, 36]; barriers also highlighted in this study. However, targeted use of Q-VAX for occupationally at-risk cohorts has proven to be cost-effective in the Australian setting, where Q fever is endemic [21]. Until a next-generation vaccine becomes available, the vaccine should be considered for similar occupationally targeted use in countries where *C*. *burnetii* is also endemic, such as the Netherlands.

Where implemented, vaccination programs should focus on increasing Q fever knowledge of at-risk cohorts, highlighting the array of species which pose a threat of *C*. *burnetii* transmission. Centralised vaccination programs through workplaces and training bodies should help to alleviate the difficulty and expense of vaccination, and promote healthy WH&S attitudes towards Q fever prevention. The vaccine should also be promoted as safe and effective to recipients and key influencers, such as veterinarians and general practitioners, who may be recommending the vaccine.

## Conclusion

This study identified that the majority of veterinarians in Australia were not willing to at least slightly recommend the Q fever vaccine to veterinary personnel across all practice types, despite vaccination being the most effective preventative measure. This finding was not unexpected given the poor uptake of Q fever vaccination among veterinary nurses and poor attitudes towards WH&S previously reported within Australia’s veterinary industry. Veterinarians need to understand their ethical and legal WH&S responsibility to reduce or eliminate the threat of hazards within the workplace, including those posed by infectious diseases such as Q fever. The findings of this study suggest that recommendation of the Q fever vaccine by veterinarians could be improved through education on Q fever, particularly the relevance of Q fever across all practice types, and improving the perception of safety of this vaccine. Improvements to vaccine uptake by veterinary nurses could be gained through education and awareness campaigns that highlight the potential for occupational exposure to *C*. *burnetii* and the importance and safety of the Q fever vaccine. Veterinary employers should aim to establish workplace practices that facilitate the vaccination process to reduce the cost or improve the perception of cost-effectiveness of this vaccine. A national approach to improving WH&S within Australia’s veterinary industry is called for, which should embrace the topic of Q fever to protect individuals and practices from the potential costs of this zoonosis when associated with serious clinical manifestations. Although specific to Q fever vaccination with Australia, the recommendations from this study are applicable for Q fever awareness programs generally and any planned introduction of the Q fever vaccine within at risk populations internationally.

## Supporting information

S1 Table Contingency table and univariable association of considered explanatory variables against the outcome variable “willing to recommend Q fever vaccination” for veterinarians surveyed in Australia in 2014. (DOC)

S2 Table Contingency table and univariable association of all considered explanatory variables against the outcome variable “sought Q fever vaccination” for veterinary nurses surveyed in Australia in 2014. (DOC)

S1 File Questionnaire administered to veterinary workers in Australia in 2014 to assess knowledge, attitudes and practices regarding Q fever disease and vaccination. (PDF)
