# Supplementary material for: Willingness of veterinarians in Australia to recommend Q fever vaccination in veterinary personnel: Implications for workplace health and safety compliance
Source: PLoS One. 2018 Jun 1;13(6):e0198421. doi: 10.1371/journal.pone.0198421 (PMC5983556; doi:10.1371/journal.pone.0198421)
Supplement: S1 Table — (DOC) [file pone.0198421.s001.doc]

**S1 Table. Contingency table and univariable association of considered explanatory variables against the outcome variable “willing to recommend Q fever vaccination” for veterinarians surveyed in Australia in 2014.**

| **Explanatory Variable** | **Willing (n)** | **Not Willing (n)** | **Total (n)** | **P-value^a^** |
| --- | --- | --- | --- | --- |
| **"I am convinced of the importance of the Q fever vaccine"** |  |  |  |  |
| Disagree | 5 | 47 | 816 | <0.001 |
| Agree | 280 | 484 |  |  |
| **"I worry that the Q fever vaccine will do more harm than good"** |  |  |  |  |
| Agree | 12 | 83 | 817 | <0.001 |
| Disagree | 275 | 447 |  |  |
| **Level of concern of personal exposure to the bacteria causing Q fever** | |  |  |  |
| Not concerned | 70 | 247 | 814 | <0.001 |
| Slightly concerned | 95 | 151 |  |  |
| Moderately concerned | 79 | 96 |  |  |
| Very concerned | 38 | 38 |  |  |
| **Level of concern that colleagues could be exposed to the bacteria causing Q fever** | | |  |  |
| Not concerned | 44 | 207 | 810 | <0.001 |
| Slightly concerned | 93 | 172 |  |  |
| Moderately concerned | 95 | 105 |  |  |
| Very concerned | 50 | 44 |  |  |
| **Level of concern that family could be exposed to the bacteria causing Q fever** | |  |  |  |
| Not concerned | 99 | 301 | 811 | <0.001 |
| Slightly concerned | 101 | 146 |  |  |
| Moderately concerned | 50 | 58 |  |  |
| Very concerned | 32 | 24 |  |  |
| **"The Q fever vaccine is safe if appropriately administered"** |  |  |  |  |
| Agree | 261 | 421 | 803 | <0.001 |
| Disagree | 2 | 20 |  |  |
| Don't know | 16 | 83 |  |  |
| **"The Q fever vaccine is too expensive"** |  |  |  |  |
| Agree | 56 | 97 | 798 | <0.001 |
| Disagree | 135 | 172 |  |  |
| Don't know | 87 | 251 |  |  |
| **"The Q fever vaccine is effective in preventing Q fever"** |  |  |  |  |
| Agree | 244 | 385 | 801 | <0.001 |
| Disagree | 2 | 5 |  |  |
| Don't know | 32 | 133 |  |  |
| **Vaccination status** |  |  |  |  |
| Vaccinated | 236 | 350 | 813 | <0.001 |
| Not vaccinated | 44 | 164 |  |  |
| Unsure | 2 | 17 |  |  |
| **Self-rated Q fever knowledge score from 1 (lowest) to 10 (highest)** |  |  |  |  |
| 1-3 | 54 | 198 | 813 | <0.001 |
| 4-5 | 69 | 146 |  |  |
| 6-7 | 103 | 128 |  |  |
| 8+ | 56 | 59 |  |  |
| **Perceived average personal level of exposure to *Coxiella burnetii* throughout career** | | |  |  |
| Don't know | 18 | 69 | 828 | <0.001 |
| Nil/very low | 64 | 162 |  |  |
| Low/moderate | 160 | 263 |  |  |
| High/very high | 45 | 47 |  |  |
| **Year of graduation from veterinary degree** |  |  |  |  |
| Prior to 1990 | 60 | 148 | 825 | 0.001 |
| 1990 - 1999 | 57 | 150 |  |  |
| 2000 - 2008 | 81 | 132 |  |  |
| 2009 onwards | 87 | 110 |  |  |
| **Personally knowing someone who has been diagnosed with Q fever** |  |  |  |  |
| No | 145 | 337 | 807 | 0.001 |
| Yes | 135 | 190 |  |  |
| **Age** |  |  |  |  |
| 18-30 | 96 | 130 | 825 | 0.005 |
| 31-38 | 72 | 122 |  |  |
| 39-49 | 56 | 148 |  |  |
| 50+ | 61 | 140 |  |  |
| **Years in total engaged in veterinary employment working directly with animals** | |  |  |  |
| 0-6 | 99 | 138 | 828 | 0.008 |
| 7-14 | 85 | 146 |  |  |
| 15-25 | 52 | 135 |  |  |
| 26+ | 51 | 122 |  |  |
| **State of current workplace** |  |  |  |  |
| NSW/ACT | 107 | 171 | 818 | 0.047 |
| Qld | 57 | 83 |  |  |
| SA/Tas/Vic | 72 | 176 |  |  |
| WA/NT | 49 | 103 |  |  |
| **Gender** |  |  |  |  |
| Male | 94 | 207 | 821 | 0.136 |
| Female | 189 | 331 |  |  |
| **Practice structure in which most hours are spent working** |  |  |  |  |
| Solo | 47 | 118 | 812 | 0.166 |
| Group | 197 | 349 |  |  |
| Corporate/Other | 32 | 69 |  |  |
| **"Q fever is a serious disease"** |  |  |  |  |
| Agree | 272 | 499 | 804 | 0.225 |
| Disagree | 4 | 15 |  |  |
| Don't know | 3 | 11 |  |  |
| **Role within practice** |  |  |  |  |
| Practice owner | 88 | 196 | 827 | 0.264 |
| Veterinary associate | 166 | 287 |  |  |
| Other | 33 | 57 |  |  |
| **"If a vaccine exists for a certain disease, then vaccination is usually a good way to protect someone against this disease"** | | | | |
| Disagree | 5 | 16 | 822 | 0.265 |
| Agree | 282 | 519 |  |  |
| **"It is difficult to get vaccinated for Q fever"** |  |  |  |  |
| Agree | 109 | 218 | 804 | 0.328 |
| Disagree | 175 | 302 |  |  |
| **University attended for veterinary school** |  |  |  |  |
| University of Sydney | 81 | 134 | 828 | 0.369 |
| University of Melbourne | 44 | 105 |  |  |
| Murdoch University | 44 | 99 |  |  |
| University of Queensland | 71 | 125 |  |  |
| Other | 47 | 78 |  |  |
| **Highest level of tertiary education attained** |  |  |  |  |
| Nil | 194 | 342 | 828 | 0.396 |
| Grad Certificate, Diploma or Masters | 47 | 107 |  |  |
| ANZCVS or equivalent, PhD or fellowship | 46 | 92 |  |  |
| **Number of staff employed by workplace** |  |  |  |  |
| 1-7 | 85 | 178 | 781 | 0.502 |
| 8-11 | 68 | 120 |  |  |
| 12-18 | 51 | 102 |  |  |
| 19+ | 69 | 108 |  |  |
| **Personally having had Q fever disease** |  |  |  |  |
| No | 248 | 474 | 814 | 0.622 |
| Yes | 34 | 58 |  |  |
| **Practice type in which most hours have been spent throughout career** | |  |  |  |
| Mixed/Large animal | 106 | 185 | 823 | 0.697 |
| Small animal | 168 | 329 |  |  |
| Other | 11 | 24 |  |  |

NSW; New South Wales. ACT; Australian Capital Territory. SA; South Australia. WA; Western Australia. NT; Northern Territory.

^a^Likelihood ratio chi-square p-value.
