# Supplementary material for: Willingness of veterinarians in Australia to recommend Q fever vaccination in veterinary personnel: Implications for workplace health and safety compliance
Source: PLoS One. 2018 Jun 1;13(6):e0198421. doi: 10.1371/journal.pone.0198421 (PMC5983556; doi:10.1371/journal.pone.0198421)
Supplement: S2 Table — (DOC) [file pone.0198421.s002.doc]

**S2 Table**. Contingency table and univariable association of all considered explanatory variables against the outcome variable “sought Q fever vaccination” for veterinary nurses surveyed in Australia in 2014.

| **Explanatory Variable** | **Vaccinated (n)** | **Not Vaccinated (n)** | **Total (n)** | **P-value^a^** |
| --- | --- | --- | --- | --- |
| **Self-rated Q fever knowledge score from 1 (lowest) to 10 (highest)** | | | |  |
| 1 | 11 | 134 | 684 | <0.001 |
| 2-3 | 47 | 190 |  |  |
| 4-5 | 65 | 107 |  |  |
| 6+ | 74 | 56 |  |  |
| **Perceived average personal level of exposure to *Coxiella burnetii* throughout career** | | | | |
| Nil/very low | 64 | 240 | 686 | <0.001 |
| Low/moderate | 100 | 103 |  |  |
| High/very high | 15 | 8 |  |  |
| Don't know | 18 | 138 |  |  |
| **"The Q fever vaccine is safe if appropriately administered"** | | |  |  |
| Agree | 184 | 305 | 677 | <0.001 |
| Disagree/Don't know | 12 | 176 |  |  |
| **"The Q fever vaccine is too expensive"** |  |  |  |  |
| Agree | 71 | 133 | 672 | <0.001 |
| Disagree | 73 | 62 |  |  |
| Don't know | 52 | 281 |  |  |
| **"The Q fever vaccine is effective in preventing Q fever"** | | |  |  |
| Agree | 166 | 288 | 678 | <0.001 |
| Disagree/Don't know | 30 | 194 |  |  |
| **Personally knowing someone who has been diagnosed with Q fever** | | | |  |
| No | 105 | 381 | 683 | <0.001 |
| Yes | 91 | 106 |  |  |
| **Practice type in which most hours have been spent throughout career** | | | |  |
| Small/equine/other | 134 | 430 |  | <0.001 |
| Large/mixed practice (traditionally associated with Q fever) | 61 | 57 |  |  |
| **State of current workplace** |  |  |  | <0.001 |
| NSW/ACT | 68 | 186 | 680 |  |
| Qld | 77 | 82 |  |  |
| SA/Tas/Vic | 36 | 143 |  |  |
| WA/NT | 17 | 71 |  |  |
| **"I am convinced of the importance of the Q fever vaccine"** | | |  |  |
| Disagree | 4 | 72 | 673 | <0.001 |
| Agree | 193 | 404 |  |  |
| **"Q fever is a serious disease"** |  |  |  |  |
| Agree | 194 | 425 | 682 | <0.001 |
| Disagree/Don't know | 3 | 60 |  |  |
| **Level of concern that colleagues could be exposed to the bacteria causing Q fever** | | | | |
| Not concerned | 50 | 215 | 684 | <0.001 |
| Slightly concerned | 58 | 146 |  |  |
| Moderately concerned | 58 | 86 |  |  |
| Very concerned | 31 | 40 |  |  |
| **Practice structure in which most hours are spent working** | | |  |  |
| Solo | 53 | 163 | 666 | <0.001 |
| Group | 92 | 272 |  |  |
| Corporate/Other | 43 | 43 |  |  |
| **Level of concern of personal exposure to the bacteria causing Q fever** | | | | 0.001 |
| Not concerned | 65 | 226 | 684 |  |
| Slightly concerned | 56 | 146 |  |  |
| Moderately concerned | 50 | 83 |  |  |
| Very concerned | 26 | 32 |  |  |
| **"It is difficult to get vaccinated for Q fever"** | |  |  |  |
| Agree | 67 | 221 | 662 | 0.001 |
| Disagree | 130 | 244 |  |  |
| **Level of influence of workplace health & safety culture regarding information about work related biosecurity** | | | | |
| Major/sole | 82 | 143 | 630 | 0.003 |
| Minor/moderate | 83 | 239 |  |  |
| Nil | 16 | 67 |  |  |
| **"I worry that the Q fever vaccine will do more harm than good"** | | |  |  |
| Disagree | 7 | 12 | 684 | 0.006 |
| Agree | 191 | 474 |  |  |
| **Level of concern that family could be exposed to the bacteria causing Q fever** | | | |  |
| Not concerned | 88 | 265 | 684 | 0.008 |
| Slightly concerned | 47 | 126 |  |  |
| Moderately concerned | 40 | 60 |  |  |
| Very concerned | 23 | 35 |  |  |
| **Level of influence of workplace protocols regarding information about work related biosecurity** | | | | |
| Major/sole | 129 | 306 | 648 | 0.062 |
| Minor/moderate | 54 | 131 |  |  |
| Nil | 3 | 25 |  |  |
| **Level of influence of AVA guidelines regarding information about work related biosecurity** | | | | |
| Major/sole | 75 | 148 | 634 | 0.085 |
| Minor/moderate | 71 | 186 |  |  |
| Nil | 36 | 118 |  |  |
| **Level of influence of vet nurses external to clinic regarding information about work related biosecurity** | | | | |
| Major/sole | 14 | 44 | 633 | 0.136 |
| Minor/moderate | 113 | 243 |  |  |
| Nil | 54 | 165 |  |  |
| **Level of influence of government sources regarding information about work related biosecurity** | | | | |
| Major/sole | 72 | 147 | 635 | 0.220 |
| Minor/moderate | 77 | 208 |  |  |
| Nil | 33 | 98 |  |  |
| **Highest level of education attained relating to veterinary nursing** | | |  |  |
| Nil | 35 | 59 | 687 | 0.236 |
| Certificate III | 13 | 30 |  |  |
| Certificate IV | 116 | 320 |  |  |
| Diploma/Bachelors/Other | 34 | 80 |  |  |
| **Level of influence of the vet nurses within clinic regarding information about work related biosecurity** | | | | |
| Major/sole | 101 | 223 | 640 | 0.237 |
| Minor/moderate | 68 | 196 |  |  |
| Nil | 12 | 40 |  |  |
| **Gender** |  |  |  |  |
| Female | 193 | 482 | 686 | 0.240 |
| Male | 5 | 6 |  |  |
| **Level of influence of veterinarians external to clinic regarding information about work related biosecurity** | | | | |
| Major/sole | 21 | 58 | 636 | 0.244 |
| Minor/moderate | 115 | 257 |  |  |
| Nil | 45 | 140 |  |  |
| **Number of staff employed by workplace** | |  |  |  |
| <6 | 42 | 137 | 670 | 0.271 |
| 6-9 | 58 | 127 |  |  |
| 10-15 | 42 | 110 |  |  |
| 16+ | 49 | 105 |  |  |
| **Years in total engaged in veterinary employment working directly with animals** | | | | |
| <5 | 49 | 131 | 681 | 0.426 |
| 5-8 | 58 | 113 |  |  |
| 9-15 | 51 | 132 |  |  |
| 16+ | 39 | 108 |  |  |
| **"If a vaccine exists for a certain disease, then vaccination is usually a good way to protect someone against this disease"** | | | | |
| Disagree | 7 | 12 | 684 | 0.452 |
| Agree | 191 | 474 |  |  |
| **Level of influence of the veterinarians within clinic regarding information about work related biosecurity** | | | | |
| Major/sole | 133 | 313 | 642 | 0.665 |
| Minor/moderate | 47 | 128 |  |  |
| Nil | 5 | 16 |  |  |
| **Age** |  |  |  |  |
| 18-26 | 56 | 142 | 680 | 0.777 |
| 27-32 | 49 | 108 |  |  |
| 33-42 | 44 | 120 |  |  |
| 43+ | 50 | 111 |  |  |
| **Level of influence of the internet regarding information about work related biosecurity** | | | | |
| Major/sole | 46 | 116 | 639 | 0.796 |
| Minor/moderate | 114 | 274 |  |  |
| Nil | 23 | 66 |  |  |
| **Level of influence of personal research through veterinary journals, textbooks and websites etc. regarding information about work related biosecurity** | | | | |
| Major/sole | 66 | 170 | 644 | 0.924 |
| Minor/moderate | 107 | 256 |  |  |
| Nil | 13 | 32 |  |  |

NSW; New South Wales. ACT; Australian Capital Territory. SA; South Australia. WA; Western Australia. NT; Northern Territory.

^a^Likelihood ratio chi-square p-value.
