# Supplementary material for: Willingness of veterinarians in Australia to recommend Q fever vaccination in veterinary personnel: Implications for workplace health and safety compliance
Source: PLoS One. 2018 Jun 1;13(6):e0198421. doi: 10.1371/journal.pone.0198421 (PMC5983556; doi:10.1371/journal.pone.0198421)
Supplement: S1 File — (PDF) [file pone.0198421.s003.pdf]

COPY OF SURVEY ADMINISTERED TO AUSTRALIAN VETERINARY WORKERS IN 2014 TO ASSESS KNOWLEDGE, ATTITUDES AND PRACTICES RELATING TO Q FEVER DISEASE AND VACCINATION

Are you currently working as a veterinarian or veterinary nurse?

- ◯ No
- ◯ Yes

Section 1

Do you intend on returning to work as a veterinarian or veterinary nurse within the next 24 months?

- ◯ No
- ◯ Yes. Please consider your most recent veterinary role when answering the questions.

Section 1

Your sex

- ◯ Male
- ◯ Female

Your age

[ ]

Your work postcode

[ ]

What is your position in your current (or most recent) workplace?

- ◯ Veterinarian
- ◯ Veterinary Nurse

Section 1

Which of the following best describes your role in your current (or most recent) workplace?

- ◯ Practice Owner
- ◯ Veterinary Associate
- ◯ General member of veterinary staff within university, government or industry
- ◯ On plant veterinarian (OPV) at an abattoir
- ◯ Other -please specify

[ ]

Section 1

At which University did you complete your veterinary degree?

- ◯ Sydney University
- ◯ Melbourne University
- ◯ Murdoch University
- ◯ Queensland University
- ◯ Charles Sturt University
- ◯ James Cook University
- ◯ Massey University New Zealand
- ◯ Adelaide University
- ◯ Other -please specify

What year did you graduate?

What is the highest level of post graduate education in veterinary science you have completed?

- ◯ No post graduate education
- ◯ Graduate certificate or diploma
- ◯ Masters Degree
- ◯ ANZCVS membership or equivalent- please specify below
- ◯ PhD or ANZCVS fellowship or equivalent- please specify below

If you have indicated "or equivalent" above please specify.

Section 1

What is the highest level of education in veterinary nursing you have completed?

- ◯ Certificate IV in Veterinary Nursing
- ◯ Diploma in Veterinary Nursing
- ◯ No formal education in Veterinary Nursing
- ◯ Other -please specify

How many years in total have you been engaged in veterinary employment where working directly with animals is part of your routine work? - include time working directly with animals in research, teaching and clinical settings

Section 1

Please estimate the number of hours per week you currently (or most recently) work directly with animals in EACH of the following veterinary environments.

Government

Corporate practice (e.g Green Cross, Banfield)

Group private practice / Multi-vet private practice

Solo private practice

Industry

Laboratory

University

Abattoir

Other- estimate hours here and specify below

Please specify "other" as indicated above.

Section 1

ONLY RESPOND TO THIS QUESTION IF YOU ARE CURRENTLY WORKING IN CLINICAL VETERINARY PRACTICE OR ARE RETURNING TO CLINICAL PRACTICE AFTER A SHORT BREAK EG MATERNITY LEAVE

Thinking of your current (or most recent) veterinary workplace where you spend the most time working directly with animals,please enter the total number of staff, including yourself.

Veterinarians

Veterinary nurses

Kennel hands, animal attendants

Administrative staff

Section 1

Thinking of your current (or most recent) veterinary workplace where you spend the most time working directly with animals, please estimate the proportion of time spent on each animal species. Please only enter whole numbers- no decimals. (responses should total 100%)

If no animal handling - Please enter 100% here.	
Dogs	
Cats	
Horses	
Dairy cattle	
Beef cattle	
Sheep	
Goats	
Pigs	
Poultry / other birds	
Pocket pets (guinea pigs, ferrets, rabbits etc)	
Fish	
Australian wildlife	
Zoo animals	
Other (please enter percentage here and specify species below)	

Please specify "other" as indicated above.

Section 1

Throughout the course of your veterinary career, on average what do you think your level of exposure to the causative agent for Q fever (Coxiella burnetii) has been?

Don't know	No exposure	Very low exposure	Low exposure	Moderate exposure	High exposure	Very high exposure
○	○	○	○	○	○	○

Section 2 - Attitudes

The next series of questions focuses on your attitudes towards the Q fever illness and Q fever vaccination. Some of the questions are general in nature while others are asking for a more personal perspective.

Thinking about vaccination for Q fever across each occupation group within each practice type, what would be your recommendations for Q fever vaccination?

Please use a scale of 1 to 7 where;

1. **Strongly recommend against vaccination**
2. **Moderately recommend against vaccination**
3. **Slightly recommend against vaccination**
4. **No recommendation either way**
5. **Slightly recommend vaccination**
6. **Moderately recommend vaccination**
7. **Strongly recommend vaccination**

VETERINARIANS

	1	2	3	4	5	6	7
Cat and dog only practice	○	○	○	○	○	○	○
Mixed practice	○	○	○	○	○	○	○
Farm animal only practice	○	○	○	○	○	○	○
Equine only practice	○	○	○	○	○	○	○
Wildlife practice	○	○	○	○	○	○	○

VETERINARY NURSES

	1	2	3	4	5	6	7
Cat and dog only practice	○	○	○	○	○	○	○
Mixed practice	○	○	○	○	○	○	○
Farm animal only practice	○	○	○	○	○	○	○
Equine only practice	○	○	○	○	○	○	○
Wildlife practice	○	○	○	○	○	○	○

KENNEL HANDS, ANIMAL ATTENDANTS AND OTHER ANIMAL HANDLERS

	1	2	3	4	5	6	7
Cat and dog only practice	○	○	○	○	○	○	○
Mixed practice	○	○	○	○	○	○	○
Farm animal only practice	○	○	○	○	○	○	○
Equine only practice	○	○	○	○	○	○	○
Wildlife practice	○	○	○	○	○	○	○

ADMINISTRATIVE STAFF WITH NO DIRECT ANIMAL HANDLING

	1	2	3	4	5	6	7
Cat and dog only practice	○	○	○	○	○	○	○
Mixed practice	○	○	○	○	○	○	○
Farm animal only practice	○	○	○	○	○	○	○
Equine only practice	○	○	○	○	○	○	○
Wildlife practice	○	○	○	○	○	○	○

Section 2

For this group of statements we are interested in your feelings and would like you to indicate your level of agreement with each of the following.

	Strongly Disagree	Disagree	Slightly Disagree	Slightly Agree	Agree	Strongly Agree
If a vaccine exists for a certain disease, then vaccination is usually a good way to protect someone against this disease	◯	◯	◯	◯	◯	◯
I am convinced of the importance of the Q fever vaccine	◯	◯	◯	◯	◯	◯
I worry that the Q fever vaccine will do more harm than good	◯	◯	◯	◯	◯	◯
It is difficult to get vaccinated for Q fever	◯	◯	◯	◯	◯	◯

Section 2

Again, thinking about your current (or most recent) veterinary workplace from a personal perspective, how concerned are you that......

	Not concerned	Slightly Concerned	Moderately Concerned	Very Concerned
…you could be exposed to the bacteria causing Q fever	◯	◯	◯	◯
...your co-workers could be exposed to the bacteria causing Q fever	◯	◯	◯	◯
... your family or your co-workers' family could be exposed to the bacteria causing Q fever	◯	◯	◯	◯

Section 2

The following statements are more general in nature, please indicate your level of agreement with each of them.

	Strongly Disagree	Disagree	Slightly Disagree	Slightly Agree	Agree	Strongly Agree	Don't know
Q fever is a serious illness with significant health consequences	◯	◯	◯	◯	◯	◯	◯
The Q fever vaccine is safe if appropriately administered	◯	◯	◯	◯	◯	◯	◯
The Q fever vaccine is effective in preventing Q fever	◯	◯	◯	◯	◯	◯	◯
The Q fever vaccine is too expensive	◯	◯	◯	◯	◯	◯	◯

Section 3 - Q fever exposure

Have you ever had Q fever?

◯ No

◯ Yes

Section 3

What year did you have Q fever? Please estimate if unsure.

How was the diagnosis made?

- ◯ Self diagnosis
- ◯ Medical practitioner -no laboratory testing
- ◯ Medical practitioner -laboratory testing
- ◯ Other -please specify

Did you take time off work as a result of your illness?

- ◯ No
- ◯ Yes

Section 3

Please indicate the number of days or weeks taken off work.

Days OR

Weeks

Section 3

Were you hospitalised during the illness?

- ◯ No
- ◯ Yes

Section 3

Please indicate the number of days or weeks that you were hospitalised.

Days OR

Weeks

To what extent did you experience each of the following:

	Did not experience	Very mild	Mild	Moderate	Severe	Very severe
Fever and chills	◯	◯	◯	◯	◯	◯
Sweats	◯	◯	◯	◯	◯	◯
Headaches	◯	◯	◯	◯	◯	◯
Muscle and joint pains	◯	◯	◯	◯	◯	◯
Fatigue	◯	◯	◯	◯	◯	◯
Endocarditis	◯	◯	◯	◯	◯	◯
Hepatitis	◯	◯	◯	◯	◯	◯
Post-Q fever fatigue syndrome	◯	◯	◯	◯	◯	◯
Pneumonia	◯	◯	◯	◯	◯	◯
Other- please indicate severity here and specify below	◯	◯	◯	◯	◯	◯

Other -please specify

____

Section 3

Do you personally know anyone who has been diagnosed with Q fever?

◯ No

◯ Yes

Section 3

How many people do you personally know who have had Q fever?

Number of people ____________________

How many of these people (that you know of) experienced a severe complication of Q fever ? eg extended time off work, endocarditis, hepatitis, post-Q fever fatigue syndrome, pneumonia

Number of people ____________________

Section 4 - Q fever vaccination

Which of the following best describes your Q fever vaccination status?

◯ I have been vaccinated

◯ I have not been vaccinated

◯ I cannot recall if I have been vaccinated

Section 4

Which of the following best describes your reason for being vaccinated for Q fever?

- ◯ I was vaccinated as part of my university course
- ◯ I was vaccinated as a requirement of my job
- ◯ I actively sought vaccination although it wasn't a specific requirement of my job or university course
- ◯ Other- please specify below

Please specify "other" as indicated above

Section 4

In what year did you receive your Q fever vaccination? Please estimate if unsure.

Please enter year as four digits eg 2002

If you are unable to estimate the year of your Q fever vaccination (above) please select "Don't know"

- ◯ Don't know

Did you experience any adverse effects after Q fever vaccination?

- ◯ No
- ◯ Yes
- ◯ Don't recall

Section 4

Were these adverse effects;

- ◯ Mild
- ◯ Moderate
- ◯ Severe

Did you seek medical attention as a result of any adverse effects of the Q fever vaccine?

- ◯ No
- ◯ Yes
- ◯ Don't recall

Section 4

Was the medical attention received from;

- ◯ General practitioner
- ◯ Hospital emergency room
- ◯ Admitted to hospital
- ◯ Other -please specify

Section 4

Before today, were you aware that there was a Q fever vaccine?

- ◯ No
- ◯ Yes

Section 4

Is the reason you have not been vaccinated for Q fever because you were ineligible as a result of pre-vaccination screening process?

- ◯ No
- ◯ Yes

Section 4

Please rate the extent to which each of the following had an influence on you not being vaccinated to date.

	No influence	Minor influence	Moderate influence	Major influence	Sole reason
I've not been able to access a service provider trained to provide Q fever vaccination	◯	◯	◯	◯	◯
The pre-screening and vaccination process is too time consuming	◯	◯	◯	◯	◯
I cannot afford the financial cost of getting vaccinated	◯	◯	◯	◯	◯
I think the Q fever vaccine may harm my health	◯	◯	◯	◯	◯
I think the Q fever vaccine may not be effective in preventing Q fever	◯	◯	◯	◯	◯
I think I won't be seriously affected by Q fever.	◯	◯	◯	◯	◯
Other - please indicate influence here and specify below	◯	◯	◯	◯	◯

Please specify "other" as indicated above.

Section 5 - Knowledge of disease risk

Please answer this group of questions based on your existing understanding of Q fever.

How would you rate your current level of knowledge of Q fever?

Please use a scale of 1 to 10 where 1 is very low (minimum) level and 10 is very high (maximum) level of knowledge.

1	2	3	4	5	6	7	8	9	10
◯	◯	◯	◯	◯	◯	◯	◯	◯	◯

Section 5

Thinking about each occupation group within each practice type in Australia, what is the level of risk of exposure to the bacterium (Coxiella burnetii) that causes Q fever ?

Please use a scale of 0 to 5 where 0 is no risk and 5 is maximum possible risk.

VETERINARIANS

	0	1	2	3	4	5
Cat and dog only practice	◯	◯	◯	◯	◯	◯
Mixed practice	◯	◯	◯	◯	◯	◯
Farm animal only practice	◯	◯	◯	◯	◯	◯
Equine only practice	◯	◯	◯	◯	◯	◯
Wildlife practice	◯	◯	◯	◯	◯	◯

VETERINARY NURSES

	0	1	2	3	4	5
Cat and dog only only practice	◯	◯	◯	◯	◯	◯
Mixed practice	◯	◯	◯	◯	◯	◯
Farm animal only practice	◯	◯	◯	◯	◯	◯
Equine only practice	◯	◯	◯	◯	◯	◯
Wildlife practice	◯	◯	◯	◯	◯	◯

KENNEL HANDS, ANIMAL ATTENDANTS AND OTHER ANIMAL HANDLERS

	0	1	2	3	4	5
Cat and dog only practice	◯	◯	◯	◯	◯	◯
Mixed practice	◯	◯	◯	◯	◯	◯
Farm animal only practice	◯	◯	◯	◯	◯	◯
Equine only practice	◯	◯	◯	◯	◯	◯
Wildlife practice	◯	◯	◯	◯	◯	◯

ADMINISTRATIVE STAFF WITH NO DIRECT ANIMAL HANDLING

	0	1	2	3	4	5
Cat and dog only practice	○	○	○	○	○	○
Mixed practice	○	○	○	○	○	○
Farm animal only practice	○	○	○	○	○	○
Equine only practice	○	○	○	○	○	○
Wildlife practice	○	○	○	○	○	○

Section 5

If an animal that was infected with the bacterium Coxiella burnetii (the cause of Q fever in humans) was presented to your clinic, please indicate the risk of transmission to someone when performing the following procedures without the implementation of any biosecurity measures (eg. PPE).

Please use a scale of 0 to 5, where 0 is no risk and 5 is maximum possible risk

	0	1	2	3	4	5
Routine physical examination	○	○	○	○	○	○
Faecal flotation	○	○	○	○	○	○
Assisting with parturition (giving birth)	○	○	○	○	○	○
Performing CPR (cardiopulmonary resuscitation)	○	○	○	○	○	○
Collecting and processing blood	○	○	○	○	○	○
Post mortem examination	○	○	○	○	○	○
Cystocentesis	○	○	○	○	○	○
Admitting the patient to hospital cage/stable	○	○	○	○	○	○
Simply being present in the room with the animal	○	○	○	○	○	○
Cleaning cages	○	○	○	○	○	○

Section 6 - Biosecurity practices

Using the broad definition of biosecurity as "a set of preventative measures designed to reduce the risk of transmission of infectious diseases" how would you rate your current level of knowledge of biosecurity as it relates to the work you undertake?

Please use a scale of 1 to 10 where 1 is very low (minimum) level and 10 is very high (maximum) level of knowledge.

1	2	3	4	5	6	7	8	9	10
○	○	○	○	○	○	○	○	○	○

Do you wash your hands before treating patients?

○ Never ○ Rarely ○ Sometimes ○ Often ○ Always

Do you wash your hands after treating patients?

○ Never ○ Rarely ○ Sometimes ○ Often ○ Always

Section 6

When performing* your usual veterinary work, what level of personal protective equipment (PPE) do you currently (or most recently) use in the following situations? - Please select all that apply.

***If you are not involved in a listed procedure, please select "Do not perform" for that procedure.**

	Do not perform	No special precautions taken	Protective clothing	Gloves	Surgical Mask	Goggles/face shield	P2/N95 respirator
Routine physical examination	☐	☐	☐	☐	☐	☐	☐
Faecal flotation	☐	☐	☐	☐	☐	☐	☐
Assisting with parturition (giving birth)	☐	☐	☐	☐	☐	☐	☐
Performing CPR (cardiopulmonary resuscitation)	☐	☐	☐	☐	☐	☐	☐
Collecting and processing blood	☐	☐	☐	☐	☐	☐	☐
Post mortem examination	☐	☐	☐	☐	☐	☐	☐
Cystocentesis	☐	☐	☐	☐	☐	☐	☐
Admitting the patient to hospital/stable	☐	☐	☐	☐	☐	☐	☐
Simply being present in the room with the animal	☐	☐	☐	☐	☐	☐	☐
Cleaning cages	☐	☐	☐	☐	☐	☐	☐

Section 6

On average how frequently does your current (or most recent) job require you to perform resuscitation ?

Never - please enter 0 ________

Times per month OR ________

Times per year ________

If yes, when resuscitating animals in general, do you use:

	Never	Rarely	Sometimes	Often	Always
Mouth to nose/mouth resuscitation	○	○	○	○	○
Breathing bag / resuscitation mask / oxygen mask / ET tube	○	○	○	○	○
Other -please select frequency here and specify below	○	○	○	○	○

Please specify "other" as indicated above

Section 6

On average how frequently does your current (or most recent) job require you to resuscitate non breathing neonatal animals (eg puppies or kittens) after caesarian birth or difficult births?

Never - please enter 0

Times per month OR

Times per year

If yes, when resuscitating non breathing puppies or kittens after caesarian birth or difficult births, do you use:

	Never	Rarely	Sometimes	Often	Always
Mouth to nose / mouth resuscitation	○	○	○	○	○
Breathing bag / resuscitation mask / oxygen mask / ET tube	○	○	○	○	○
Other, please select frequency here and specify below	○	○	○	○	○

Please specify "other" as indicated above

Section 6

Which of the following information sources are your main source of influence regarding information about work related biosecurity?

	No influence	Minor influence	Moderate influence	Major influence	Sole influence
Protocols established by the employer/practice	○	○	○	○	○
Veterinarians within your practice	○	○	○	○	○
Veterinarians outside your practice	○	○	○	○	○
Veterinary nurses within your practice	○	○	○	○	○
Veterinary nurses outside your practice	○	○	○	○	○
My personal research through veterinary journals and textbooks, websites etc	○	○	○	○	○
My personal research through the internet	○	○	○	○	○
Australian Veterinary Association Biosecurity Guidelines	○	○	○	○	○
Government or Health authority	○	○	○	○	○
Workplace culture	○	○	○	○	○
Other- please select influence here and specify below.	○	○	○	○	○

Please specify "other" as indicated above

Section 6

What is your level of responsibility within your workplace with respect to workplace health and safety (WHS).

Please use a scale of 1 to 5 where 1 is not at all and 5 is completely responsible.

	1	2	3	4	5
Training of other staff in WHS in my workplace	○	○	○	○	○
Ensuring that staff comply with WHS requirements or established protocols in my workplace	○	○	○	○	○
Establishing WHS protocols for my workplace	○	○	○	○	○
Other - please select level here and specify below	○	○	○	○	○

Please specify "other" as indicated above

THANK YOU FOR TAKING THE TIME TO COMPLETE THE QUESTIONNAIRE. YOUR PARTICIPATION IS APPRECIATED.

After you submit the questionnaire you will be redirected to the prize draw for an iPad. You can choose whether you would like to enter this. Please note that this is done via a separate link and will not be linked to your responses to this questionnaire.

IF YOU WOULD LIKE TO OBTAIN MORE INFORMATION ABOUT Q FEVER OR Q FEVER VACCINATION PLEASE VISIT THE FOLLOWING WEBSITES.

Australian Government Department of Health and Ageing- The Australian Immunisation Handbook 10th Edition 2013
www.health.gov.au

CSL Q Vax-R Q fever vaccine
www.csl.com.au
